# Image of ‘‘sudden block by the iris’’ before completion of traumatic aniridia: indirect partial support for Navon’s hypothesis

**DOI:** 10.1186/s12886-025-04280-9

**Published:** 2025-07-28

**Authors:** Atsuhide Takesue, Shintaro Nakao

**Affiliations:** 1 Department of Ophthalmology, Juntendo Nerima Hospital, 3-1-10 Takanodai, Nerima-ku, Tokyo, 177-8521 Japan; 2 Department of Ophthalmology, Juntendo University Graduate School of Medicine, Tokyo, Japan

**Keywords:** Traumatic aniridia, Blunt trauma, Surgical wound, Surgical peripheral iridectomy

## Abstract

**Background:**

Traumatic aniridia mainly occurs following blunt trauma to the eyeball. Navon hypothesized that ‘‘sudden block by the iris’’ was a necessary step in the development of traumatic aniridia, but this has not been confirmed.

**Case presentation:**

The patient was a man in his 70s who sustained a right eye injury in a fall onto an umbrella stand. Slit lamp examination of the right eye revealed brownish spherical tissue in the anterior chamber, which was one-third filled with hyphema, and brownish pigmentation was observed beneath the conjunctiva. The next morning, following a dramatic decrease in the hyphema, it was surprising to find that none of the previously observed spherical tissue was present and the iris was absent around the whole circumference. The entire iris had seemingly been expulsed under the conjunctiva via an old surgical wound.

**Conclusions:**

The photograph in this case might show the ‘‘sudden block by the iris’’ hypothesized by Navon almost 30 years ago. The photograph may be one piece of indirect and partial evidence that supports ‘‘sudden block by the iris occurs as a precursor to traumatic aniridia’’.

## Background

Traumatic aniridia mainly occurs as a result of blunt trauma to the eyeball or a perforating wound. The iris completely separates from the ciliary body, and then the detached iris escapes through a prior surgical wound or a new traumatic corneal or scleral wound. Consequently, the iris is absent from the anterior chamber and the ciliary processes are visible. Traumatic aniridia is often discovered with surprise and puzzlement following the absorption of hyphema from the anterior chamber. Most cases occur after cataract surgery, but some occur after trabeculectomy or suture for ocular trauma.

In 1997, Navon hypothesized a possible mechanism of traumatic aniridia [1]: ‘‘(1) The force of trauma transiently distorted the cataract incision, causing it to leak. (2) Aqueous outflow created a lifting action over the iris that drew it to plug the wound by the Bernoulli effect [a phenomenon in fluid dynamics where the pressure in a fluid decrease as its velocity increases]. (3) This sudden block in aqueous flow created a pressure gradient across the tunnel sufficient to disinsert the iris and deliver it through the wound. (4) Renewed outflow of iris and aqueous depressurized the eye, which prevented extension of the wound or creation of new rupture sites.’’.

Interestingly, he hypothesized that the ‘‘sudden block by the iris’’ was a necessary step in the occurrence of traumatic aniridia. While several case reports [2–4] have cited his hypothesis, its correctness remains uncertain. Presumably because no doctors have witnessed the process of traumatic aniridia unfolding. In the present report, a clinical photograph is shared that might show the proposed ‘‘sudden block by the iris” just before the completion of traumatic aniridia.

## Case report

**Fig. 1:**
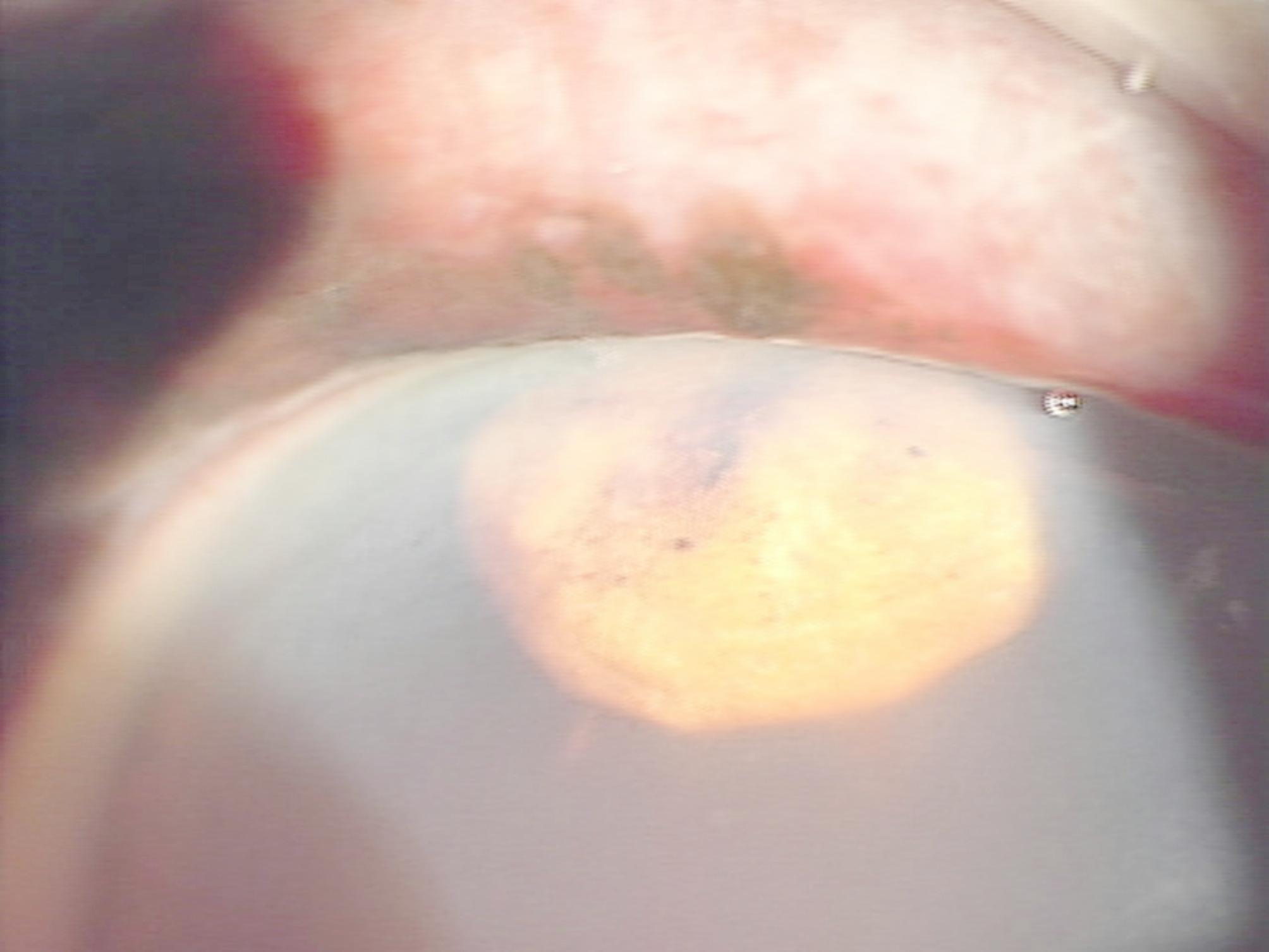
Clinical photograph showing brownish spherical tissue in the anterior chamber

The patient was a man in his 70 s who sustained a right eye injury in a fall onto an umbrella stand. At presentation, there was no obvious tear in the ocular surface. His best corrected visual acuity (BCVA) was hand motion OD and 6/8.5 OS. Intraocular pressure (IOP) was 24 mmHg OD and 10 mmHg OS. Slit lamp examination of the right eye revealed brownish spherical tissue in the anterior chamber (Fig. 1), more than one-third of which was filled with hyphema, and brownish pigmentation was seen beneath the conjunctiva with subconjunctival hemorrhage at the superior-temporal corneal limbus. Because the brownish pigmentation was adjacent to the brownish spherical tissue in the anterior chamber and he had undergone surgical peripheral iridectomy to prevent acute glaucoma attack 47 years earlier, a part of the iris presumably was expelled through the dehisced surgical wound. Magnetic resonance imaging showed that the eyeball shape was maintained and there was no foreign body or hematoma in the orbit.

**Fig. 2:**
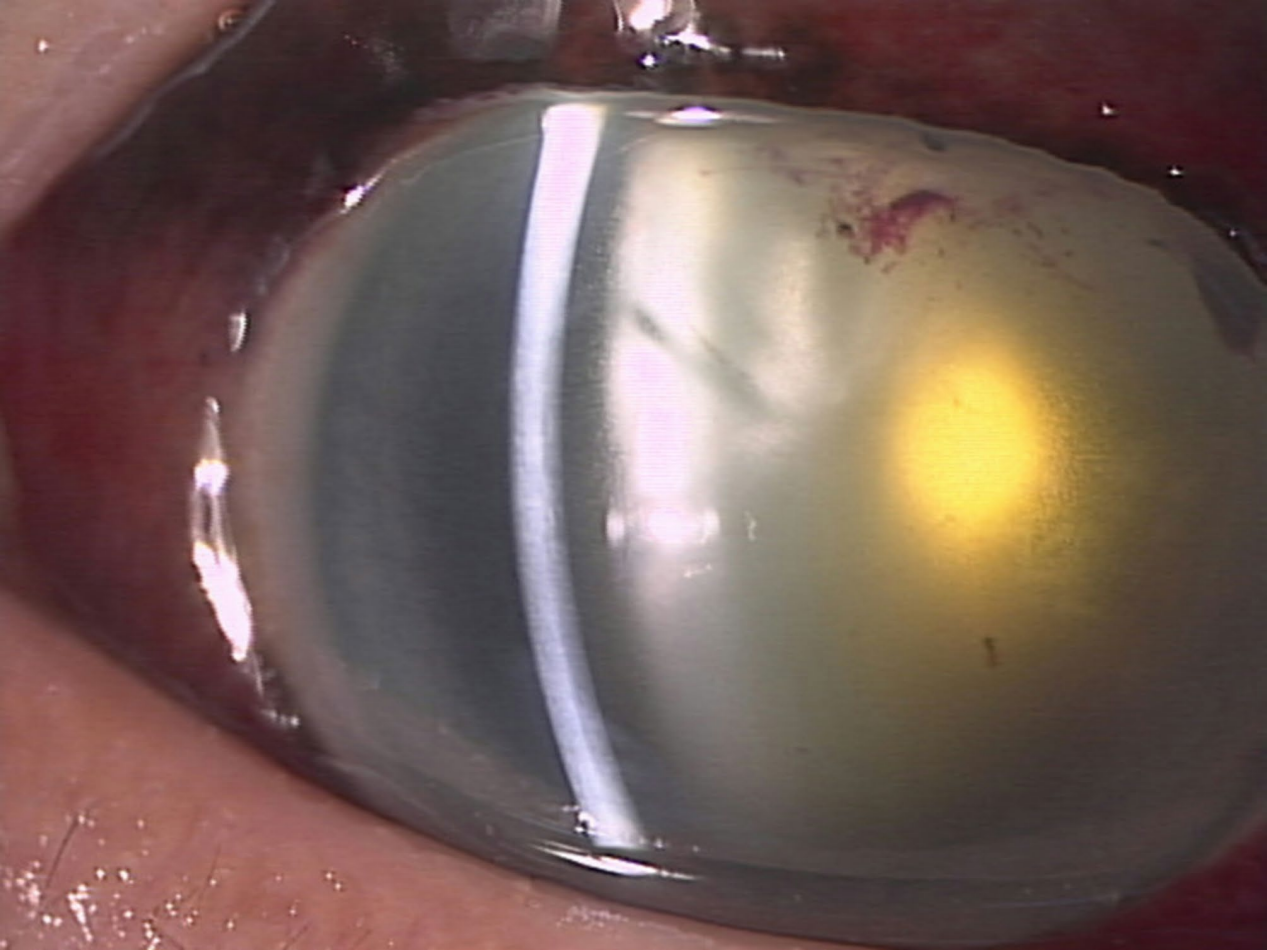
Clinical photograph showing that the spherical tissue had spontaneously been expulsed via the surgical wound by the next morning

The next morning, following a dramatic decrease in the hyphema, it was surprising to find that the iris tissue had disappeared, and there was 360° loss of iris (Fig. 2).　The entire iris and most of the blood had seemingly been expulsed under the conjunctiva via the old surgical wound. IOP was the same as before (24 mmHg OD). That evening, the conjunctiva was incised, the prolapsed iris removed, and the old surgical wound sutured. It was then confirmed that there were no other scleral ruptures.

**Fig. 3:**
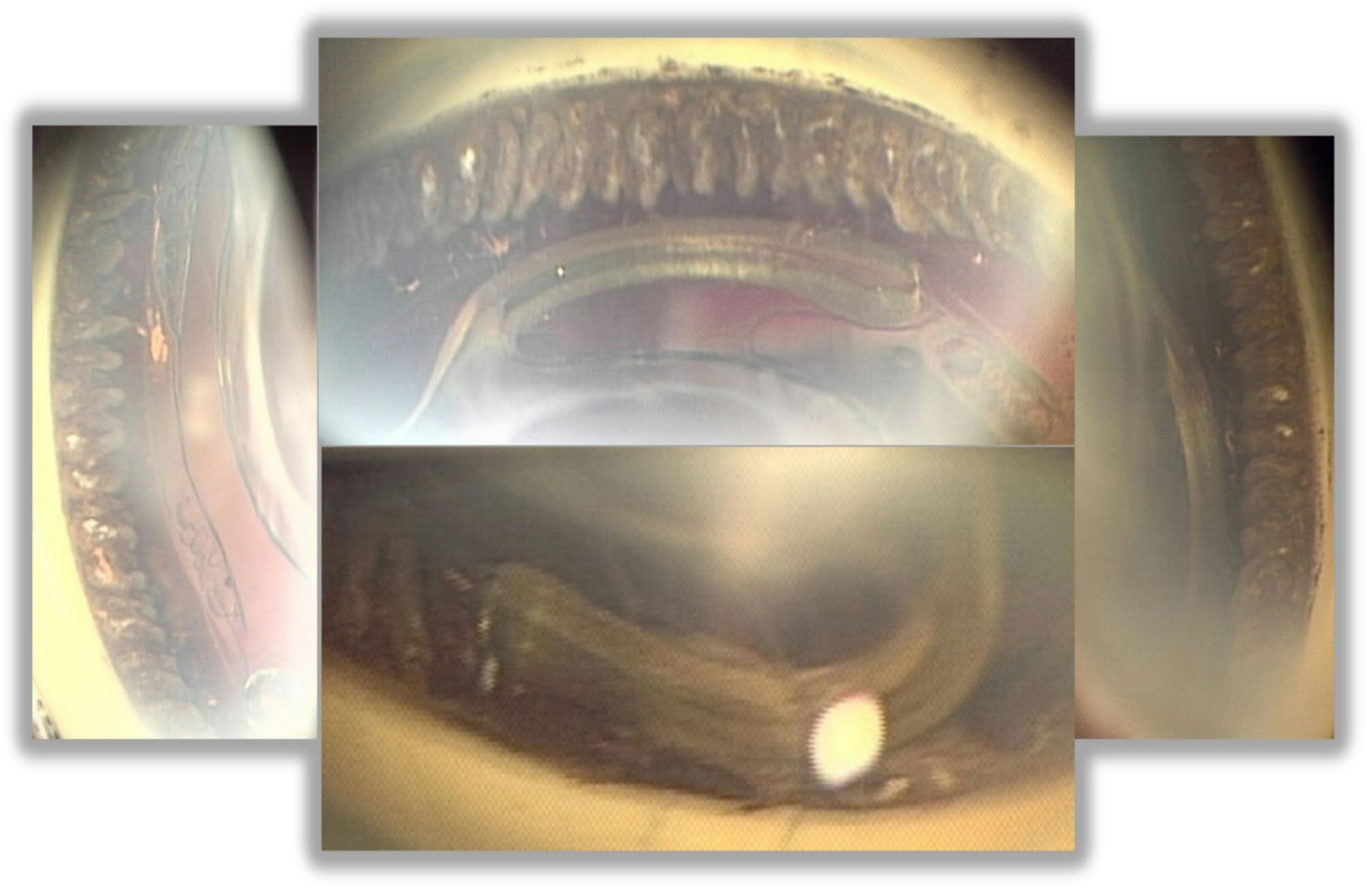
Clinical photographs using a gonioscope. Due to the aniridia, the ciliary body that had undergone photocoagulation can be directly observed

Once the IOP stabilized 17 days later, cataract surgery was performed. All the zonular fibers were intact, so the lens was in place and stable although the anterior chamber remained very shallow during the surgery due to the absence of the iris. Postoperatively, the high IOP of 25 mmHg persisted even after applying various types of glaucoma eye drops. Therefore, photocoagulation was applied to the ciliary body (wavelength 577 nm, power 200 mW, duration 0.5 s, total 81 spots) based on general knowledge of endoscopic cyclophotocoagulation. As the shape of the ciliary body could be directly observed owing to the aniridia, a laser lens could be substituted in selective laser trabeculoplasty (Fig. 3). The IOP normalized within 2 weeks. Due to preexisting macular degenerative disease, BCVA has not improved more than 6/60. Although a black diaphragm intraocular lens was not implanted, the patient has not complained of photophobia postoperatively.

## Discussion

While most of the cases of traumatic aniridia occur within several years after cataract surgery, a case has been reported in which it occurred 30 years afterward [5]. In the present case, traumatic aniridia occurred 47 years after surgical peripheral iridectomy.

Although it was surprising during the cataract surgery to find that all the zonular fibers were intact and, therefore, that the lens was in place and stable, this finding seems to be well known. Several case reports have noted intact zonular fiber after traumatic aniridia [1, 2, 6, 7].

When the patient was first examined, no iris prolapse had occurred, and it was not until the following day that this could be confirmed. In Navon’s hypothesis, iris prolapse was thought to occur continuously immediately after injury, and other previous reports have not discussed the time from injury to completion of iris prolapse. However, at least in this one case, it took several hours for iris prolapse to be completed. It is unclear whether this was because the impact of the injury was weak or because the old surgical wound did not open wide due to adhesions.

Reported cases of traumatic aniridia can be classified into three types. (1) In total traumatic aniridia, the iris is completely missing over 360°of the ciliary body. The iris is not present in the anterior chamber, having been expulsed via the wound. This type accounts for almost all reported cases. (2) In non-expulsed isolated aniridia, the iris is missing over 360° of the ciliary body, but the detached iris settles in the inferior anterior chamber [2]. (3) In partial traumatic aniridia with partial iris expulsion, part of the iris is missing and expulsed from the anterior chamber, while part remains intact on the ciliary body [4]. If the case reported in this previous study [4] had subsequently and spontaneously developed into total traumatic aniridia, the photograph of ‘‘Partial traumatic aniridia with partial iris expulsion’’ might have been an image of ‘‘sudden block by the iris’’ that is thought to lead to total traumatic aniridia.

The present case had total traumatic aniridia, but more importantly, it could be photographed as it unfolded, just 1 day before its completion. This photograph (Fig. 1) is believed to represent the ‘‘sudden block by the iris’’ proposed by Navon almost 30 years ago [1]. If so, to the authors’ knowledge, this photograph may be the world’s first to demonstrate that ‘‘sudden block by the iris’’ occurs, at least in some cases, as a precursor to traumatic aniridia. It should also be acknowledged that this report is based on only one case and is a retrospective observation, so its interpretation is limited. The present static image is insufficient to demonstrate the dynamic sequence proposed in Navon’s theory. Future research is expected to understand the mechanisms of traumatic aniridia.

### Limitation

Navon hypothesized that the sequence is: ① positive pressure (impact), ② negative pressure, ③ positive pressure (sudden block), ④ negative pressure (completion of traumatic aniridia). This case report was written on the assumption that Navon’s hypothesis was correct, but it may be possible that sudden block is not necessary to complete traumatic aniridia in the first place. In other words, there might be a possibility that traumatic aniridia can be achieved by steps ① and ② alone. If this is the case, it is possible that the iris that was incarcerated into the wound in the photograph was in the middle of process ②.

## Conclusion

Navon hypothesized that ‘‘sudden block by the iris’’ was a necessary step in the process of developing traumatic aniridia. The photograph in this case may be one piece of indirect and partial evidence that supports ‘‘sudden block by the iris occurs as a precursor to traumatic aniridia’’.

## Data Availability

No datasets were generated or analysed during the current study.
